# ART initiation in an outpatient treatment center in Dakar, Senegal: A retrospective cohort analysis (1998-2015)

**DOI:** 10.1371/journal.pone.0202984

**Published:** 2018-09-19

**Authors:** Ndeye Fatou Ngom, Mame Awa Faye, Kiné Ndiaye, Aminata Thiam, Cheikh Tidiane Ndour, Jean-François Etard, Papa Salif Sow, Moussa Seydi, Eric Delaporte, Amandine Cournil

**Affiliations:** 1 Ambulatory Treatment Center, Fann Hospital University, Dakar, Senegal; 2 University Alioune Diop of Bambey, Diourbel, Senegal; 3 Department of Infectious diseases, Fann Hospital University, Dakar, Senegal; 4 Transvihmi, UMI 233-Institut de Recherche pour le Developpement, U1175-Inserm, University of Montpellier, Montpellier, France; The Ohio State University, UNITED STATES

## Abstract

**Objective:**

To examine how patient characteristics combined with ART eligibility expansions affect the initiation of antiretroviral therapy (ART) among eligible patients attending a referral center in Senegal from 1998 to 2015.

**Methods:**

This is a retrospective observational study carried out at the outpatient treatment Centre (Centre de Traitement Ambulatoire) in Dakar, Senegal, based on computerized medical records, gathered from 1998 to 2015, of ART-naïve patients over 15 years of age. ART eligibility was defined as (CD4 count below 200) or as (WHO stage 4) or as (WHO stage 3 with (CD4 count below 350 or with unavailable CD4 count)) in 1998–2010; as (CD4 count below 350) or as (WHO stage 3 or 4) in 2011–2013; as (CD4 count below 500) or as (WHO stage 3 or 4) in 2014–2015. Four periods were defined according to ART eligibility expansions and Senegal’s HIV care history: 1998–2003 (P 1), 2004–2010 (P 2), 2011–2013 (P3), and 2014–2015 (P4). Patients were expected to participate financially in their treatment during the first period (P1).

**Results:**

A total of 3651 patient records were included. The median patient age was 40 years (IQR: 32–48). Women represented 56% of the population. The median CD4 count was 183 cells/mm^3^. Overall, 53% of patients had CD4 < 200 cells/mm^3^ at entry. This proportion reached 45% in 2014–2015. 2535 patients (69%) were eligible for therapy, including 1503 (41%) who started ART. The proportion of treated patients among those who were eligible at entry or later increased steadily from 25%, 47%, 75% to 82% in the four periods, respectively. The median time to treatment decreased from 5.6 months (IQR: 3–11) in P1 to 0.8 months (IQR: 0–2) in P4. Eligible patients with more advanced disease (CD4<200 cells/mm^3^ and/or clinical stage 3 or 4) were more likely to be ART initiated than those with CD4≥200 cells/mm^3^ and/or clinical stage 1 or 2 at each stage of ART eligibility expansion.

**Conclusion:**

ART eligibility expansions were marked by a sharp increase in the proportion of eligible patients initiating treatment. These results show that in terms of management, the target of "Test and Treat" can be easily reached but that HIV testing will remain a key element to improve treatment success, as illustrated by the high proportion of people with advanced stage of infection at the time of ART initiation.

## Introduction

Despite significant progress increasing access to HIV treatment in recent years, many regions in sub-Saharan Africa continue to see high rates of attrition between the time of testing and diagnosis, to linkage to care and initiation of antiretroviral therapy (ART) [1]. The reason for long delay between linkage to care and ART initiation is the cumbersome process of identifying and then readying patients eligible for ART. This process often involves long waiting times and numerous clinical visits requiring the initiation of the medical file, biological assessment of the CD4 count, evaluation of patient eligibility, and pretreatment educational sessions, all of which must take place prior to ART [2,3].

In September 2015, WHO issued new guidelines for HIV care, advocating for the immediate treatment of all infected persons, regardless of the level of CD4 count [4]. This "Treat-all" or "Test and Treat" strategy follows previous changes in minimum CD4 counts required for initiation of ART, which increased from 200 to 350 cells/mm^3^ in 2010, to counts < 500 cells/mm^3^ in 2013 [5,6]. The latest recommendation encourages ART delivery in an increasingly short time frame, even immediately after confirmation of the diagnosis of HIV infection, simplifying the process ART initiation and thus reducing the attrition rate. It is a key strategy for achieving the second target of the "90-90-90" targets in 2020 [7].

Senegal HIV prevalence rate is very low and stable in the general population around 0.7% to 0.5% according to the latest UNAIDS 2016 data [8]. However, the prevalence rate is significantly higher in key-populations (17.8% in men having have sex with men, 6.6% in sex workers, 9.4% in people who inject drugs and 2% in prisoners [9–12]. In addition, the number of patients newly infected have decreased by 50% between 2001 and 2016 [8]. Female are 1.6 times more infected than male regardless of age group. With regards to treatment, only 52% of HIV-positive people in Senegal received ARV treatment in 2016 [9]. The Programme National de Lutte contre le SIDA (PNLS) was established to coordinate the government’s anti-AIDS activity in 1986 and was later renamed the Conseil National de Lutte contre le SIDA (CNLS). CNLS’s goals for 2030 include zero new infections, zero deaths tied to AIDS, and zero discrimination against HIV-positive Senegalese citizens. Senegal, like most of the international community, adopted UNAIDS "90-90-90" strategy when establishing TATARSEN (Test All, Treat All and Retain) in 2016. Senegal’s long experience in antiretroviral drugs use, dating back to 1998 and which has followed the evolution of various changes in therapeutic recommendations proposed by WHO, provides an excellent opportunity for evaluating the consequences of changing recommendations in order to better understand and anticipate obstacles to achieving the second "90", i.e. 90% of all people with diagnosed HIV infection receiving ART.

The aim of the present study was to examine how patient characteristics combined with ART eligibility expansions affect the initiation of ART among eligible patients attending a referral center in Senegal from 1998 to 2015.

## Methods

### Study setting

The Ambulatory Treatment Center (CTA) at Fann Hospital in Dakar, Senegal, specializes in the comprehensive care of people living with HIV. It was created in 1998 thanks to a tripartite collaboration between the Pan-African Organization for the Fight against AIDS, the French Red Cross and the Ministry of Health of Senegal. It is one of the first three centers for therapeutic management of people living with HIV started under the Senegalese Initiative for Access to Antiretrovirals (ISAARV), the first governmental initiative in Africa.

### Sources of data

In 2011, the ESTHER (Ensemble pour une Solidarité Thérapeutique Hospitalière en Réseau) hospital partnership initiative has supported the implementation of ESOPE, an electronic medical software system used to monitor the care of people living with HIV, at several care sites in Africa, including the Dakar CTA. This software makes it easier to manage individual patients by creating electronic records of their data that can be easily accessed, searched or shared between treatment centers. ESOPE also provides a powerful means of evaluating HIV / AIDS treatment programs through the automated production of synthetic reports. As of 2012, medical records of all new CTA patients were entered into the software. At the same time, a retrospective entry of all the medical files since 1998 has been carried out, thus expanding the electronic database that can be used for evaluation. The database was also cleaned and updated with active search of people lost-to follow-up prior to the data extraction used for the analysis presented here.

### Population analyzed (inclusion criteria)

Included in this analysis are HIV-infected patients with an ESOPE file opened between August 1, 1998 and December 31, 2015, who were naïve of ART at entry and who were at least 15 years of age. The analyses were run using all visits from August 1, 1998 until June 30, 2016. Patients registered as outpatients, because they were monitored at another center, were excluded from the analysis.

### Variables and definitions

The data used in this analysis included patient age, sex, marital status, occupation, place of residence, body mass index (BMI), WHO clinical stage of infection, type of HIV, presence of tuberculosis, presence of HbS antigen. These data were recorded at entry. CD4 count at entry (first visit) and at all subsequent visits and date of initiation of ART were also used. Marital status at entry was recorded as single, married (polygamous or monogamous), widowed or divorced. Occupation at entry was classified as unemployed, self-employed or salaried job. Place of residence at entry was categorized in four regions (City of Dakar, Center, North, South-east). BMI was computed as weight/ height^2^ (kg/m^2^) and categorized according to WHO classification (underweight, <18.5; normal, 18.5–24.9, overweight, 25–29.9 and obese, ≥30). Date of initiation of ART was defined as the date of the visit during which any antiretroviral regimen (excluding treatment taken for prevention of mother-to-child transmission) was prescribed for the first time in ART-naïve subjects.

In this analysis, ART eligibility was defined according to WHO guidelines:

- as CD4 count below 200 regardless of clinical criteria or as WHO stage 4 regardless of CD4 count or as WHO stage 3 with CD4 count below 350 or with unavailable CD4 count from 1998 to 2010 in accordance with WHO guidelines published on November 30, 2003 [13] and January 1, 2006 [14];
- as CD4 count below 350 regardless of clinical criteria or as WHO stage 3 or 4 regardless of CD4 from 2011 to 2013 in accordance with WHO guidelines published on July 15, 2010 [5].;
- as CD4 count below 500 regardless of clinical criteria or as WHO stage 3 or 4 regardless of CD4 count from 2014 to 2015 in accordance with WHO guideline published September 30, 2013 [6].

Clinical conditions such as tuberculosis or hepatitis B co-infections, pregnancy were not taken into account to define eligibility in this analysis because these informations were not consistently recorded in the database.

Four different time periods were defined according to Senegal’s HIV care history and WHO ART eligibility expansions. Period 1 (P1), 1998–2003 which is characterized by the start of the Senegalese Initiative for Access to Antiretrovirals (ISAARV). During this time patients were expected to participate financially in their treatment (on the basis of a scale established after a socio-economic survey); and Period 2 (P2) 2004–2010; Period 3 (P3) 2011–2013 and Period 4 (P4) 2014–2015 based on WHO guidelines publication and ART eligibility expansions for the last two periods.

The patient population is divided into four groups based on treatment eligibility and whether they had started ART:

- **Eligible and started on ART**—patients who were eligible for treatment at entry into care or became eligible during follow-up and who had started treatment while attending CTA;
- **Eligible but not started on ART**—patients who were eligible for treatment at entry into care or became eligible during follow-up but who did not start treatment while attending CTA;
- **Not eligible and not started on ART**—patients who remained ineligible for starting ART during the duration of their attendance at CTA;
- **Not eligible but started on ART**—patients treated before eligibility. This group corresponds to individuals for whom the initiation of treatment was decided on the basis of criteria (such as AIDS stage during follow-up, co-infections (tuberculosis, hepatitis B), pregnancy, serodiscordance, belonging to key populations, viral load, etc.) that were not taken into account in our definition of eligibility. This group represents less than 10% of the population analyzed and is not integrated into the group of treated patients for the sake of simplification.

For eligible patients, the **time delay** was defined by the duration, in months, between eligibility and the start of treatment (for treated patients) or between eligibility and the last follow-up visit (for untreated patients).

**Pre-ART visits** (missed opportunities) correspond to follow-up visits occurring after a patient became eligible for treatment but prior to the onset of ART. The visit in which treatment is initiated is not counted as a pre-ART visit.

### Statistical analysis

Population characteristics and treatment modalities were compared by Chi-2 tests for categorical variables or by non-parametric variance analyzes (Kruskall-Wallis) for continuous variables.

Factors associated with treatment initiation among eligible patients were identified using a Cox model that took into account the delay in initiating treatment from the date the subject is eligible. Patients who were not put on treatment during follow-up were censored at the date of the last follow-up visit to the center. The choice of the variables to include in the model was based on the literature and their availability in the ESOPE database. The interaction terms between the time periods and the different variables were tested in order to identify the covariates whose effects varied with time. As several interactions with time periods were identified at a 0.05 p-value threshold., models were built separately for each time period. The model selection was based on a backward selection using a p-value threshold of 0.20. In the models the variable age was dichotomized to <40 and ≥40 years old, 40 years being the median. The association with region was assessed by comparing Dakar to any other regions because sample size was reduced in some regions. As clinical stage and CD4 count were not independent, the association with clinical stage and CD4 level is assessed using a single variable that divides the eligible population into 5 groups: clinical stage 1 or 2 and CD4 ≥200 cells/mm^3^; clinical stage 3 or 4 and CD4 ≥200 cells/mm^3^; clinical stage 3 or 4 and CD4 not measured; clinical stage 1 or 2 and CD4 <200 cells/mm^3^; and clinical stage 3 or 4 and CD4 <200 cells/mm^3^.

The STATA 14.0 software (Stata Corporation LP, College Station, TX, USA) was used for data analysis. P-values of less than 0.05 were considered significant for comparisons.

### Regulatory ethical considerations

The analysis involved anonymized data and thus did not contain the identity or exact address of individuals. The opening of the medical record, follow-up, and ART initiation were done with consent of the patient. The protocol was approved by the Ethics Committee of Senegal.

## Results

### Population analyzed

**Fig 1:**
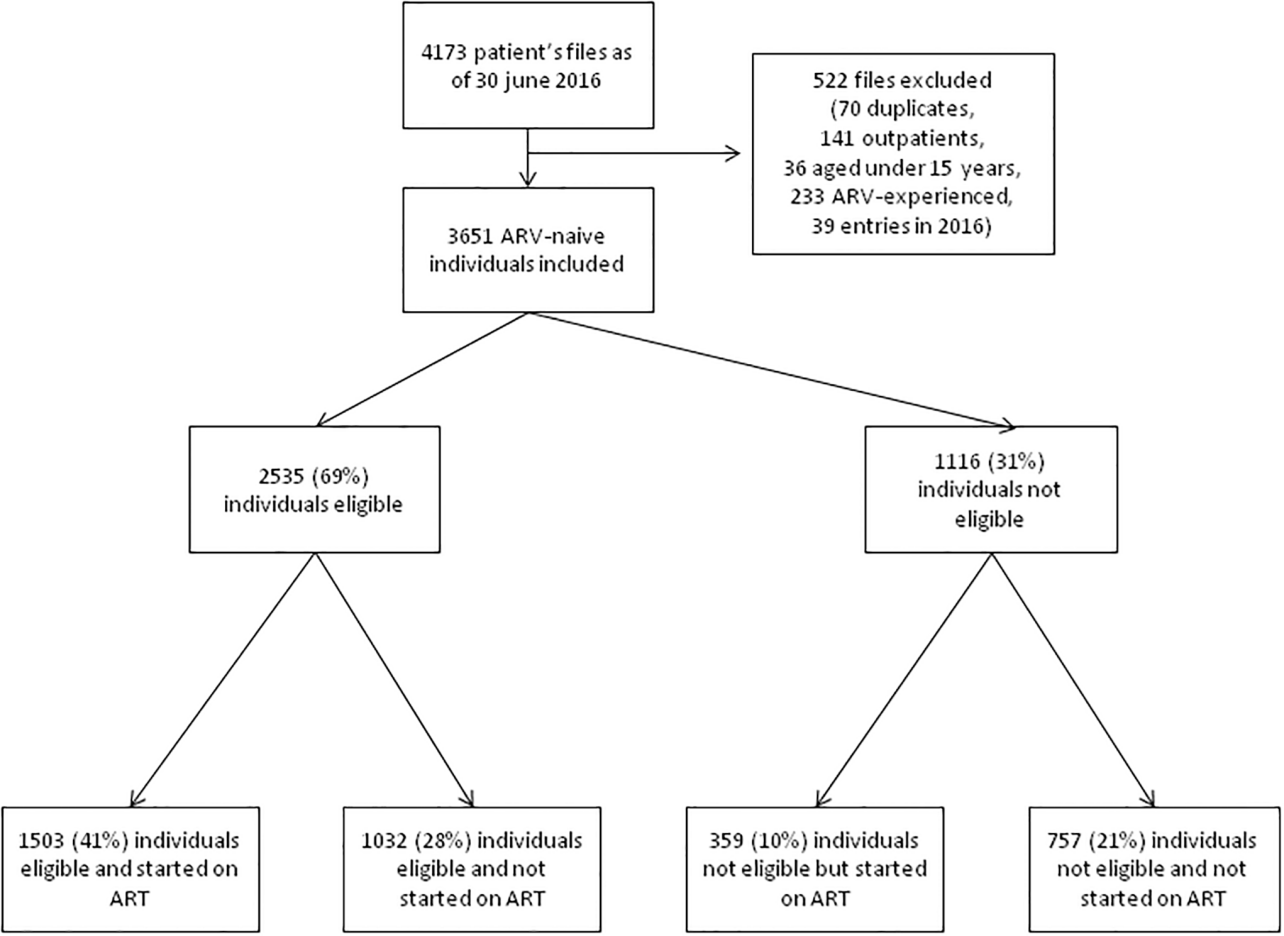
Selection of the study population and distribution according to eligibility at entry into care or later and treatment initiation during the follow-up in CTA.

As of June 30, 2016, the ESOPE database had 4173 files associated with 51076 visits. We excluded a total of 522 patient files, corresponding to 70 duplicates, 141 outpatients, 36 patients under 15 years of age, 233 patients who were not ART naïve at the opening of the case, 39 patients with files opened between 30 December 2015 and 30 June 2016, and 3 cases for which no visits had been recorded. The population analyzed thus contains 3651 ART-naïve patients for whom a file was opened at CTA between 1998 and 2015 (Fig 1).

**Fig 2:**
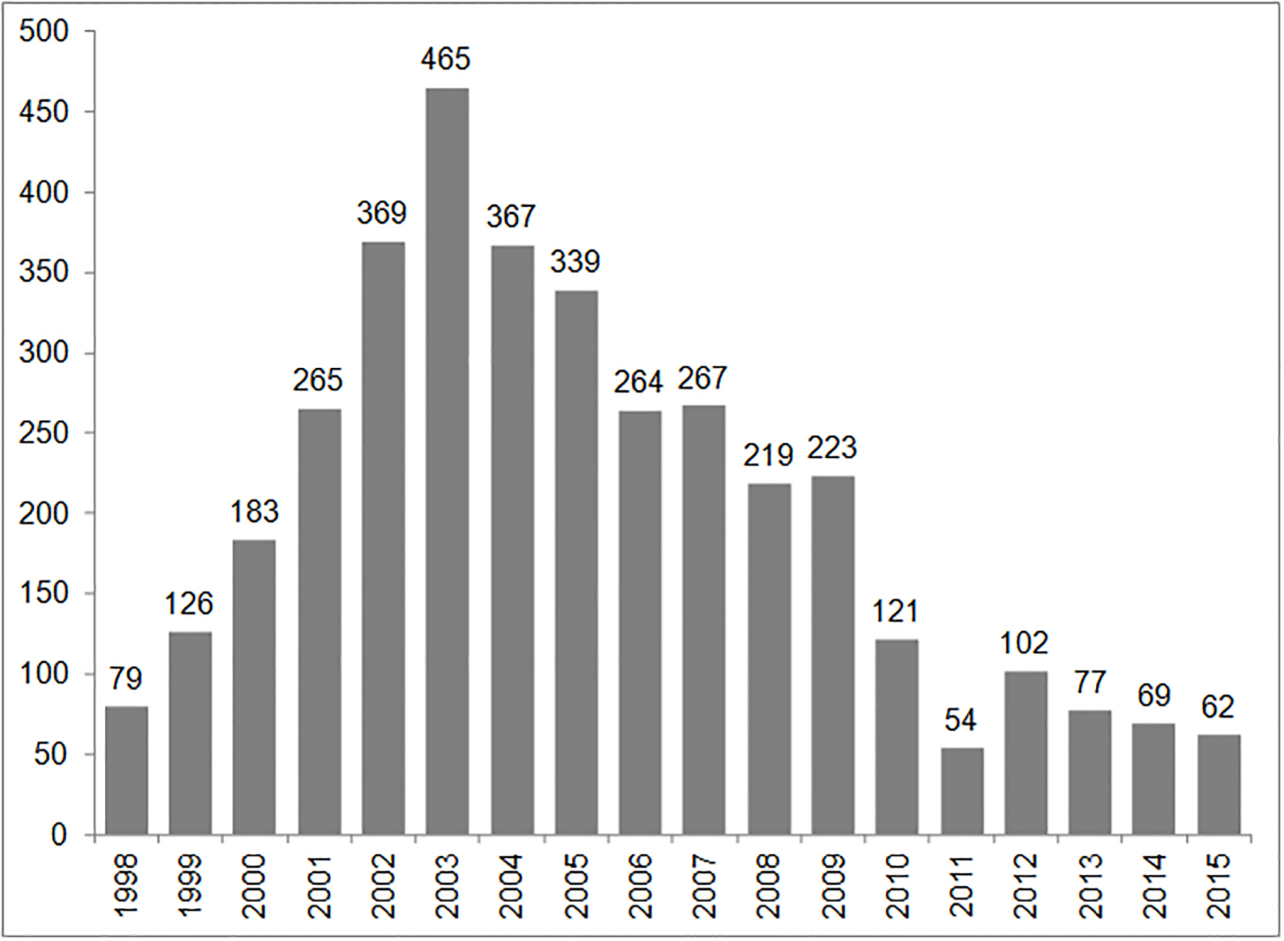
Number of entries of ARV-naïve patients by year, CTA, Dakar, Senegal.

The annual number of entries increased gradually from 1998 to a peak in 2003, when 465 new cases were opened. This figure has since decreased gradually and has been around 70 new patients per year since 2013 (Fig 2).

### Characteristics of naïve patients at entry

The median patient age at entry over the entire study period was 40 years (Table 1). The median age decreased over time. Over the study period, 56% of patients were women, peaking at 59%% in the period 2004–2010. There was a change in the distribution of the reported marital status of patients, with an increase in the proportion of single persons, who represented 27% of the population in the last period and a significant decrease in the proportion of widowers over time. The proportion of WHO stage 1 patients at entry into care increased from 11% in the first period to 34% in the last period. However, the proportion of patients in stages 3 and 4 remained high in the last period, representing almost half of the patient population. There was a significant improvement in the availability of CD4 counts for evaluation at entry: while only 42% of people had a CD4 count at the opening of their file in the first period, this proportion increased to 92% in the last period. The median CD4 count increased slightly over time (169 [68–307] in 1998–2003 and 240 [97–398] in 2014–2015, p-value = 0.02). The proportion of people with CD4 <200 cells/mm^3^ still accounted for 45% of the population with available CD4 in the last period.

**Table 1:** Characteristics of individuals at entry.

	Periods (at entry)				Total
	1998–2003	2004–2010	2011–2013	2014–2015	
	n = 1487	n = 1800	n = 233	n = 131	n = 3651
**Age, median [IQR], years**	41 [34–50]	39 [31–46]	37 [29–46]	39 [28–47]	40 [32–48]
**Age groups (years) %**, ***					
15–29	12.8	19.0	27.0	28.2	17.3
30–39	30.3	34.2	30.5	23.7	32.0
40–49	31.7	30.8	26.2	31.3	30.9
above 50	25.1	16.0	16.3	16.8	19.8
Sex (% female)**	53.8	59.4	53.2	49.6	56.4
Marital Status %***					
Single	15.9	17.5	32.6	27.5	18.2
Married	55.1	55.9	46.3	45.0	54.6
Widowed	18.1	13.7	7.7	9.2	14.9
Divorced	10.8	12.8	13.3	18.3	12.2
Occupation %***					
Unemployed	36.1	28.8	27.5	16.8	31.2
Self-employed	57.8	61.1	66.1	74.8	60.6
Salaried job	6.1	10.1	6.4	8.4	8.2
Region of residence %***					
Dakar	80.4	89.0	93.1	96.9	86.0
Center	16.7	8.3	6.9	3.1	11.4
North	1.5	2.0	0.0	0.0	1.6
South-East	1.4	0.7	0.0	0.0	0.9
WHO clinical stage %***					
1	11.0	18.1	24.5	34.3	16.2
2	49.2	34.1	13.7	16.8	38.3
3	34.9	35.6	35.2	30.5	35.1
4	4.9	12.2	26.6	18.3	10.4
**Body mass index, n**	**1324**	**1712**	**204**	**119**	**3359**
**Groups %, kg/m**^2^***					
<18.5	38.5	40.4	34.3	32.8	39.0
18.5–25	50.3	47.5	47.6	47.0	48.7
> 25	11.2	12.1	12.1	20.2	12.3
Tuberculosis co-infection %***	17.0	15.1	6.9	6.9	15.0
**HIV type %**					
HIV-1	89.6	90.6	95.7	93.9	90.6
HIV-2	7.0	7.6	3.9	6.1	7.1
HIV-1 &- 2	3.4	1.8	0.4	0.0	2.3
**CD4 cell count, n**	628	1635	205	121	2589
**CD4 cell count, median [IQR], cells/mm**^3^**	168 [68–307]	186 [58–361]	204 [56–417]	240 [97–398]	183 [63–356]
**Groups %, CD4 cells/mm**^**3**^ ***					
<200	57.6	52.7	49.3	44.6	53.3
200–349	21.8	20.6	19.0	20.7	20.1
350–499	10.5	12.7	15.1	15.7	12.4
≥500	10.0	13.9	17.6	19.0	13.5

P-values for comparison between time-periods:

*** <0.0001,

**<0.001.

### Eligibility and treatment according to periods

Out of a total of 3651 naïve patients, 2535 were eligible for treatment according to treatment criteria as defined. The vast majority of eligible patients were so at entry, with 420 patients becoming eligible at follow-up.

**Fig 3:**
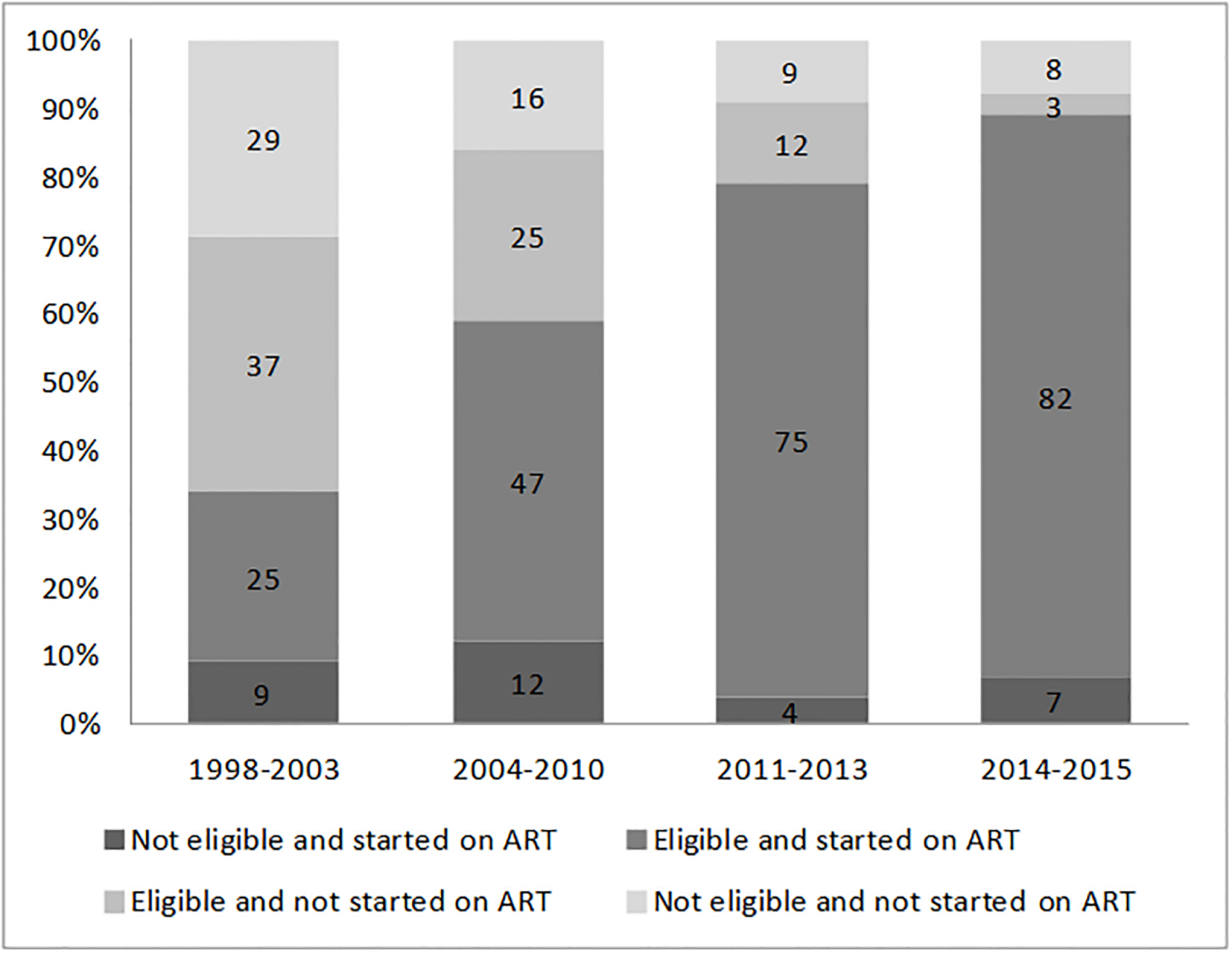
Distribution of study population according to eligibility at entry into care or later, treatment initiation during the follow-up in CTA and period at entry.

As expected, given ART eligibility expansion, the proportion of eligible patients increased significantly over time, from 62% in the first period to 85% in the last (Fig 3). Among eligible naïve patients, treatment was initiated in 1503 cases (59%), with a progressive increase more than doubling during the period reviewed, from 39% in period 1 to 88% in period 4.

Of the non-eligible patients, 359 had initiated ART during follow-up before eligibility. This group represents 10% of the population studied.

### From eligibility to ART initiation or loss to follow-up: Delay and number of pre-ART visits

Over the entire study period, the median time to ART was 2.0 months, with a significant decrease from 5.6 months to 0.8 months between the first and last periods (Table 2). In the last period, 78% received treatment within 3 months after documented eligibility, whereas it represented only a quarter of the patients in the first period. The reduction in the time to treatment initiation was not accompanied by a reduction in the number of pre-ART visits after becoming eligible. The proportion of people put on treatment immediately after becoming eligible increased little over time, from 5% to 9% between the first and last periods. The proportion of eligible patients making at least 5 pre-ART visits after becoming eligible was stable between the first and last periods (11% to 12%).

**Table 2:** Delay and number of pre-ART visits from eligibility to ART initiation or to loss to follow-up among eligible subjects.

Eligible subjects, n	Periods (at eligibility)				
	1998–2003	2004–2010	2011–2013	2014–2015	Total
	858	1277	227	173	2535
**Started on ART, n (%)**	**336 (39%)**	**811 (64%)**	**203 (89%)**	**153 (88%)**	**1503 (59%)**
Median delay [IQR], months	5.6 [3.0–10.8]	1.9 [1.0–3.8]	0.6 [0.3–1.9]	0.8 [0.4–2.0]	2.0 [0.8–5.4]
Delay %, months					
< 3	89 (26.5)	557 (68.3)	165 (81.3)	119 (77.8)	927 (61.7)
3–5	89 (26.5)	124 (15.3)	7 (3.4)	12 (7.8)	232 (15.4)
6–11	83 (24.7)	66 (8.1)	13 (6.4)	11 (7.2)	173 (11.5)
≥ 12	75 (22.3)	67 (8.3)	18 (8.9)	11 (7.2)	171 (11.4)
Median number of visits [IQR]	2 [1–3]	1 [1–2]	1 [1–4]	2 [1–4]	1 [1–3]
Number of visits, %					
0	16 (4.8)	23 (2.8)	12 (5.9)	14 (9.1)	65 (4.3)
1	104 (30.9)	465 (57.3)	95 (46.8)	60 (39.2)	724 (48.2)
2 to 4	178 (53.0)	264 (32.6)	63 (31.0)	60 (39.2)	565 (37.6)
≥ 5	38 (11.3)	59 (7.3)	33 (16.3)	19 (12.4)	149 (9.9)
**Not started on ART, n (%)**	**522 (61%)**	**466 (36%)**	**24 (11%)**	**20 (12%)**	**1032 (41%)**
Median delay [IQR], months	2.4 [0.2–6.9]	1.3 [0.0–3.9]	0.3 [0.0–3.0]	4.4 [0.0–13.2]	1.8 [0.0–5.3]
Delay months, %					
< 3	287 (55.0)	325 (69.7)	19	10	641 (62.1)
3–5	92 (17.6)	71 (15.2)	0	0	163 (15.8)
6–11	72 (13.8)	29 (6.2)	1	4	106 (10.3)
≥ 12	71 (13.6)	41 (8.8)	4	6	122 (11.8)
Median number of visits [IQR]	3.0 [2.0–4.0]	2.0 [1.0–3.0]	2.0 [1.0–5.0]	3.0 [1.0–4.0]	3.0 [1.0–4.0]
Number of visits, %					
1	126 (24.1)	118 (25.3)	9	6	259 (24.0)
2 to 4	285 (54.6)	285 (61.2)	8	10	588 (57.4)
≥ 5	111 (21.3)	63 (13.5)	7	4	185 (18.5)

Among eligible patients, 41% did not initiate treatment while attending CTA. About one quarter did not return to the center after the visit at which eligibility was documented. Overall, 57% went for consultation between 2 to 4 times, and 19% had 5 or more visits between eligibility and completion of follow-up.

### Factors associated with treatment in eligible individuals

Eligible subjects over 40 years old were less likely to be treated compared with subjects below 40 years during the first period (1998–2003) (Table 3). The age effect was not evidenced in the other periods. The association between region of residence and treatment was observed in 2004–2010; people living in Dakar were more likely to be treated. Occupation and marital status showed varying associations with treatment initiation in the different periods of time.

**Table 3:** Factors associated with ART initiation among eligible subjects.

		Periods (at eligibility)							
		Period 1 1998–2003 858		Period 2 2004–2010 1277		Period 3 2011–2013 227		Period 4 2014–2015 173	
		aHR	95% CI	aHR	95% CI	aHR	95% CI	aHR	95% CI
Age, years									
≥ 40 vs <40		0.7**	[0.6;0.9]	0.9	[0.8;1.1]				
Sex									
Female vs male						0.7	[0.5;1.0]		
Occupation									
Salaried job vs unemployed				1.2	[1.0;1.6]	0.7	[0.4;1.3]		
Self-employed vs unemployed				1.2*	[1.0;1.4]	0.7*	[0.5;1.0]		
Marital Status									
Married vs single		1.5*	[1.0;2.3]	0.9	[0.7;1.1]	1.4	[0.8;2.3]		
Widowed vs single		1.1	[0.8;1.4]	0.8*	[0.7;1.0]	1.5*	[1.1;2.1]		
Divorced vs single		1.0	[0.7;1.4]	1.0	[0.8;1.3]	1.3	[0.7;2.3]		
Region									
Dakar vs other regions		1.2	[0.9;1.7]	1.3*	[1.0;1.7]				
Clinical stage & CD4 count at eligibility, cells/mm^3^									
1 or 2	≥ 200					ref.		ref.	
3 or 4	≥ 200	ref		ref.		2.7***	[1.6;4.4]	2.5**	[1.3;4.6]
3 or 4	missing	0.7	[0.5;1.2]	0.7*	[0.5;1.0]	0.8	[0.5;1.4]	3.1*	[1.4;6.9]
1 or 2	<200	2.0**	[1.2;3.1]	2.4***	[1.8;3.0]	4.5***	[2.4;8.2]	2.6**	[1.5;4.5]
3 or 4	<200	1.5	[0.9;2.5]	2.9***	[2.3;3.7]	3.1***	[2.1;4.6]	3.5***	[2.3;5.3]

aHR–adjusted Hazard Ratio, CI–Confidence Interval.

Covariates are recorded at entry unless otherwise specified.

P-values for comparison between time-periods:

*** <0.001,

**<0.01,

* <0.05.

ART eligibility was defined as CD4 count below 200 regardless of clinical criteria or as WHO stage 4 regardless of CD4 count or as WHO stage 3 with CD4 count below 350 or with unavailable CD4 count from 1998 to 2010; as CD4 count below 350 regardless of clinical criteria or as WHO stage 3 or 4 regardless of CD4 from 2011 to 2013; as CD4 count below 500 regardless of clinical criteria or as WHO stage 3 or 4 regardless of CD4 count from 2014 to 2015.

The influence of disease progression, measured by taking into account both the CD4 count and the clinical stage, was observed at all periods. Within eligible subjects, those who presented with CD4<200 cells/mm^3^ regardless of clinical stage were more likely to be treated compared to those with CD4≥200 cells/mm^3^ with a clinical stage 3 or 4 in the first two periods from 1998 to 2010. In periods 3 and 4, in which ART eligibility expands to CD4 <350 and to <500 cells/mm^3^, patients with CD4<200 were also more likely to be treated compared to those with CD4≥200 cells/mm^3^ and clinical stage 1 or 2 as well as patients with CD4≥200 cells/mm^3^ and clinical stage 3 or 4, with smaller risk, however.

Table 4 indicated that patients with CD4<200 were also treated more rapidly with shorter delay between eligibility and ART initiation (Table 4).

**Table 4:** Median delay from eligibility to ART initiation according to HIV disease progression among eligible subjects.

Median [IQR] delay, months		Periods (at eligibility)			
		Period 1 1998–2003	Period 2 2004–2010	Period 3 2011–2013	Period 4 2014–2015
Clinical stage at entry & CD4 count (cells/mm^3)^ at eligibility		336	811	203	153
1 or 2	≥ 200	-	-	1.4 [0.5–7.5]	1.5 [0.5–7.2]
3 or 4	≥ 200	11.9 [4.8–21.2]	3.9 [1.7–13.9]	0.5 [0.3–1.2]	0.5 [0.3–1.8]
3 or 4	missing	9.6 [5.3–16.1]	4.1 [1.6–27.3]	1.2 [0.1–12.2]	0.9 [0.5–1.4]
1 or 2	<200	3.9 [2.6–7.6]	1.9 [1.1–3.4]	0.3 [0.2–0.6]	0.5 [0.2–0.8]
3 or 4	<200	4.9 [2.9–9.3]	1.5 [0.9–2.8]	0.5 [0.2–1.1]	0.5 [0.2–1.6]

## Discussion

Change in the demographic profile of the ART-naïve patients attending the CTA from 1998 to 2016 was marked by a decline in the median age of the population. This trend cannot be explained by a demographic shift in the epidemics in Senegal as the proportion of young people (15–24 years) among new HIV cases decreased over time from 34% in 1998 to 24% in 2017 [15]. This observation may be attributed to the implementation of active screening of young people who are highly vulnerable to HIV infection, especially young people belonging to key risk groups such as men who have sex with men and sex workers.

The degree of immunosuppression characterizing our study population at entry into care was relatively constant over time. For period 1, nearly 58% of files had missing CD4 count data, making interpretation of the median value difficult. From period 2 until the end of the study period, the median CD4 counts increased slightly over time but remained low in the last period (2014–2015) with a median of 240 [97–398] cells/mm^3^ and 45% of patients with CD4 count below 200 cells/mm^3^. This means that most HIV-infected persons present to care at CD4 counts far below the applicable threshold at this time (i.e. <500 cells/mm^3^). A meta analysis of 56 studies from Sub-Saharan Africa found no significant change in CD4 count at entry into care from 2002 to 2013, with a mean CD4 count of 309 cells/mm^3^ (95% confidence interval, 237–381 cells/mm^3^) in 2012 [16]. In 2018, an analysis of IeDEA and COHERE database including 951855 patients from 55 countries showed that median CD4 cell count at ART initiation increased in all countries with the smallest increase observed in West-African countries (118 to 186 cells/mm^3^) [17].

The high proportion of immuno-suppressed subjects at entry into care can be explained both by a late diagnosis and an entry delayed by several months or even years after the diagnosis. This situation is observed in most countries, including in countries with high resources [18–22]. In this analysis we were unable to document late screening and delayed entry into care because the date of first testing was not consistently recorded in the medical file.

Efforts to increase ART coverage, the most important of which was to provide free access to care, resulted in an increase from 39% of eligible patients started on ART in period 1 to 88% in period 4. This favorable development was facilitated by the steady decline in the annual number of ART-naïve people arriving at the clinic from 2003 onward.

This improvement in initiation of ART is observed in other countries notably Kenya, where a study found that among eligible patients, the percentage those starting ART increased from 29% in 2007 to 61% in 2012 [23]. A meta-analysis of programmatic data from 22 countries examined the changes in timely ART initiation after ART eligibility criteria were expanded to CD4≤350 and CD4≤500. This study showed that ART eligibility expansions were followed by substantial increases in rates of timely ART initiation after enrollment in the clinic. Larger increases were observed among newly eligible patients but significant increases were also observed among previously eligible patients as shown in the present study [24]. Another study conducted in rural KwaZulu-Natal, South Africa also reported large increases in ART initiation within 3 months of programme entry from 2007 to 2012 [25]. A meta-analysis of 8 studies conducted between 2002 and 2010 found an overall proportion of 63% of eligible people initiating treatment, a figure comparable to 64% of ART coverage of eligible patients in 2004–2010 in the present study [26]. These results are a consequence of the commitment of the international community and countries in the fight against HIV with free access to treatment, the WHO 3x5 initiative, and universal access. However, efforts to improve access to treatment centers and treatment are still needed in resource-limited countries. According to the latest UNAIDS report, while globally 54% [40–65%] of adults aged 15 years old and older accessed treatment, in sub-Saharan Africa, this value falls to 36% [27].

We observed that the increase in the proportion of patients initiating ART was accompanied by a reduction in the time to treatment from 5.6 months in the first period to less than 1 month in the last period (0.8 months). Moreover, the majority of patients (76%) initiated their treatment within three months after eligibility. These results are similar to those observed in other studies. A study in India [28] found that 67% of eligible patients started treatment within three months after eligibility and in Uganda, 64.5% began treatment three months after eligibility, for the period 2007–2011[29].

The sharp decrease in the time between eligibility and ART that we observed may reflect the fact that while Senegal was committed early on to providing ART, changes in the ability of patients to easily access care and in provide treatment improved significantly over time. Initially, the principle of a financial contribution was adopted and the process of accessing treatment required a socio-economic survey. The complete dossier was then presented to the eligibility committee, who then decided whether or not to start ART as well as determining the expected financial contribution of the patient. Moreover, during this period, financial constraints often led to stock-outs for CD4-testing reagents and ARVs, which delayed the start of treatment for many patients [30].

We showed that among eligible people, those with more advanced disease were more likely to initiate timely treatment. This was observed at all periods, with stronger effects after ART eligibility criteria expanded to CD4<350 and CD4<500. These findings showed that in the clinic, ART eligibility expansions did not affect negatively ART initiation among those with more advanced diseases. These findings are consistent with other studies and meta-analysis, showing large increases of ART initiation among newly eligible patients with less advanced disease, without any decrease of rate of ART initiation among those with low CD4 count or advanced clinical stage (24,25). This prioritization is likely due to the influence of WHO recommendations and programs advising caregivers in resource-constrained countries to prioritize the most highly immunosuppressed patients, in whom delays in initiation of ART is associated with high risk of mortality. Unlike what has been observed in a systematic review of African studies, we did not find neither a sustainable effect of age nor an effect of sex indicating that men and young people were less likely to initiate ART after enrollment into care [31].

This work has several limitations. The information in the medical file was entered into ESOPE at the time of the patient visit only since 2012; all visits prior to 2012 were retrospectively entered, resulting in a loss of some information and missing data for the first periods. The definition of eligibility used in this analysis did not take into account specific situations, such as pregnant women, persons living with a partner who are serodiscordant, men who have sex with men, sex workers, or drug users for whom treatment is routinely offered regardless of CD4 count or clinical stage. It also did not take into account the passage to a higher clinical stage during follow-up. Lastly, the analysis focused on data from a referral treatment center specializing in the management of HIV, meaning that results cannot be generalized across the country’s various HIV care structures.

The strength of this work lies in the fact that it was carried out in one of the largest reference centers in Senegal, which has seen patients throughout the historical periods of therapeutic treatment of HIV since 1998. Today, CTA is the only center in Senegal, set up 12 years after the start of therapeutic management, with a computerized medical monitoring tool of the patient. Significant resources were deployed to ensure the retrospective entry of patient data prior to 2012 and in follow-up that enabled us to carry out this work.

## Conclusions

Since 1998, ART eligibility expansions were associated with tremendous improvement in the rate of ART initiation and significant reduction in the delay to treatment among eligible patients at the CTA of Fann with no adverse impact on ART initiation among those with advanced disease who remained prioritized regarding access to ART.

These results show that in terms of management, the target of "test and treat" can be easily achieved but that timely diagnosis and linkage to care remain key elements in ensuring positive outcomes, as illustrated by the lack of improvement in patients initiating care when presenting in an advanced stage of infection.

More generally, improved targeting and testing combined with the application of the latest WHO recommendations for the immediate treatment of any infected person should limit prioritization practices and contribute to improving antiretroviral coverage to achieve the goals of "90-90-90" and ultimately help to end the epidemic.
